# Pharmacological Treatments of Negative Symptoms in Schizophrenia—An Update

**DOI:** 10.3390/jcm13185637

**Published:** 2024-09-23

**Authors:** Evangelia Maria Tsapakis, Michael Treiber, Calypso Mitkani, Zoe Drakaki, Anastasios Cholevas, Cleanthe Spanaki, Konstantinos N. Fountoulakis

**Affiliations:** 1 3rd Department of Psychiatry, Aristotle University of Thessaloniki, 54636 Thessaloniki, Greece; 2 Department of Neurosciences, School of Medicine, University of Crete, 71003 Heraklion, Greece; 3 Department of Psychiatry and Psychotherapy, Medical University of Vienna, 1090 Vienna, Austria; 4 Comprehensive Center for Clinical Neurosciences and Mental Health (C3NMH), Medical University of Vienna, 1090 Vienna, Austria; 5 Department of Neurology, Agios Pavlos General Hospital of Thessaloniki, 55134 Thessaloniki, Greece; 6 Department of Neurology, University Hospital of Heraklion, Voutes, 71110 Crete, Greece

**Keywords:** schizophrenia, negative symptoms, pharmacological treatment

## Abstract

Schizophrenia is a chronic psychotic disorder comprising positive symptoms, negative symptoms, and cognitive deficits. Negative symptoms are associated with stigma, worse functional outcomes, and a significant deterioration in quality of life. Clinical diagnosis is challenging despite its significance, and current treatments offer little improvement in the burden of negative symptoms. This article reviews current pharmacological strategies for treating negative symptoms. Dopaminergic, glutamatergic, serotonergic, noradrenergic, cholinergic, anti-inflammatory compounds, hormones, and psychostimulants are explored. Finally, we review pharmacological global treatment guidelines for negative symptoms. In general, switching to a second-generation antipsychotic seems to be most often recommended for patients with schizophrenia on first-generation antipsychotics, and an add-on antidepressant is considered when depression is also present. However, the treatment of negative symptoms remains an unmet need. Future, larger clinical studies and meta-analyses are needed to establish effective pharmacological agents for the effective treatment of negative symptoms.

## 1. Introduction

Schizophrenia is a chronic psychotic disorder affecting both individuals and society. Dopaminergic dysfunction, reduced glutamatergic neurotransmission, and increased proinflammatory status play central roles in symptom development, but serotonergic and cholinergic dysfunction may also be present. Its symptoms can be distinguished into three main categories: positive, negative, and cognitive. Positive symptoms, including delusions, hallucinations, and thought disorders, are a crucial part of the disease; negative and cognitive symptoms, however, are more closely related to the decline in quality of life and poor functional outcomes. Females have been shown to have lower rates of negative but higher rates of depressive and anxious symptoms. They also experience a three-year earlier average disease onset than males [1]. A 20-year follow-up study of FEP patients found that male sex and longer duration of untreated psychosis predicted the presence of negative symptoms, which were continuous and unaffected by treatment and associated with lower neurocognitive function and a higher use of antipsychotic medication [2]. Importantly, negative symptoms also manifest in adolescents and children in the early stages of psychosis before diagnostic criteria for schizophrenia are met [3].

According to the National Institute of Mental Health’s Measurement and Treatment Research to Improve Cognition in Schizophrenia (NIMH-MATRIS) consensus statement, the main domains of negative symptoms are blunted affect, alogia (reduction in the quantity of words spoken), avolition (reduced goal-directed activity due to decreased motivation), asociality, and anhedonia (reduced experience of pleasure) [4,5,6]. Anhedonia was described as early as 1956 as a core genetic phenotype of schizophrenia [7]. Interestingly, self-reported anhedonia is thought to be present before the onset of psychosis and is linked to a high risk of developing the disorder. Furthermore, trait anhedonia persists throughout the course of schizophrenia and is associated with poor social, vocational, and cognitive functioning [8].

Negative symptoms can be quantified and assessed using multiple approaches, including (a) the Scale for the Assessment of Negative Symptoms (SANS), (b) the Positive and Negative Syndrome Scale (PANSS), (c) the Brief Psychiatric Rating Scale (BPRS), (d) the Negative Symptom Assessment (NSA-16), (e) the Brief Negative Symptom Scale (BNSS), and (f) the Clinical Assessment Interview for Negative Symptoms (CAINS) [9]. Negative symptoms can occur at any time during the illness [10]. However, clinical detection remains challenging as most patients and carers may not report negative symptoms, leading clinicians to focus on detecting and assessing the “noisier” positive symptoms and signs of relapse, hostility, or suicidality.

Moreover, significant correlations may exist across the five negative symptom domains, which do not necessarily share similar neurobiological substrates and could represent separate therapeutic targets. Two key differences have been identified regarding negative symptom aetiopathogenesis. Firstly, the five domains can be classified into experiential (avolition, asociality, and anhedonia) and expressive (alogia and blunted affect) dimensions, with researchers generally agreeing, however, that studies on negative symptoms should take a nuanced approach, addressing all five domains [11]. Secondly, it is important to distinguish between primary and secondary negative symptoms, where the latter are thought to be due to other factors, including positive symptoms, medication side effects, social factors, or comorbid disorders such as depression, as secondary negative symptoms may respond to different treatment strategies [10,12]. A recent study using network analysis successfully distinguished between depressive and negative symptoms [13]. However, the crucial distinction between primary and secondary negative symptoms is often overlooked in clinical trials and research on the underlying causes of these symptoms [14,15,16]. Hence, effective pharmacological treatment of negative symptoms remains an unmet clinical need.

Negative symptoms have also been linked to neuroimaging abnormalities. The ENIGMA consortium’s meta-analytic results indicate an association between overall negative symptoms and reduced cortical thickness in the medial orbitofrontal cortex [17]. Although evidence supporting the link between negative symptoms and reduced ventrolateral prefrontal cortex (VLPFC) activity is limited, more consistent findings have emerged regarding the ventral striatum. A recent meta-analysis including data from 23 studies found a significant association between negative symptoms and reduced activation in the left ventral striatum. This area of the brain is associated with reward anticipation, which is believed to be involved in the development of anhedonia and avolition [18]. Some studies suggest that this association may be specific to experiential negative symptoms. However, more extensive studies are needed for confirmation as many studies included in this meta-analysis did not differentiate between primary and secondary negative symptoms, and hypoactivation of the ventral striatum was also correlated with positive symptoms, raising concerns about “pseudo-specificity” [19]. The longitudinal trajectories of negative symptoms in individuals with FEP and their association with changes in brain cortical thickness were recently investigated in a follow-up study of 357 FEP patients over ten years [18]. Three dimensions of negative symptoms were revealed, namely, expressivity, experientiality, and attention. Trajectory analyses identified three patterns of symptom progression: stable, decreasing, and increasing. Participants with an increasing trajectory in the expressivity dimension showed cortical thinning in specific frontal regions from the third to the tenth year after symptom onset. The study also found that the areas of cortical thinning overlapped with regions of lower receptor density, suggesting a potential link between cortical abnormalities and molecular profiles. These findings suggest that stable and decreasing trajectories are typical, while cortical thickness abnormalities in the expressivity dimension may serve as a biomarker for poor symptom outcomes [18].

## 2. Materials and Methods

This selective review aims to provide a broad overview of recently obtained data on pharmacological strategies that are either in development or under investigation to target negative symptoms in schizophrenia.

The research question was to evaluate the efficacy of all medications in the treatment of negative symptoms. To define the research question, we followed the patient population, intervention, comparator, outcome (PICO) framework [20]. We chose adults with schizophrenia spectrum disorder, treatment with any drug or placebo or FGAs, and negative symptom assessment scales. 

MEDLINE was searched for articles written from inception to 29/2/2024 that matched any combination of the following keywords: ((negative symptoms schizophr* AND (dopa-min* OR glutam* OR antiinflammat* OR seroton* OR noradren* OR anticonvuls* OR psychostim* OR hormones OR anticholinerg*)) AND treat*) and limited to the English language, Humans, and Reviews. We also checked the references in the relevant publications we found to acquire any additional data, as well as the official sites of the pharmaceutical companies developing the novel compounds in question. Lastly, we reviewed treatment guidelines from around the globe on treating negative symptoms of schizophrenia, focusing on treatment with pharmacological agents.

## 3. Results

Our MEDLINE search initially yielded 2251 results—there were 2129 articles restricted to the English language and 1629 to Humans. When limited to Reviews, 522 articles were retrieved in total. The eligibility assessment of the studies was implemented by authors EMT and MT separately, and the final decision for inclusion was discussed with KNF.

### 3.1. The Dopamine (DA) System 

A recent meta-analysis of MRI brain alterations in schizophrenia with persistent negative symptoms found functional alterations to be predominantly concentrated in the thalamocortical and default mode networks [21], partially confirming current pathophysiological models of experiential negative symptoms suggesting an aberrant communication between sub-cortical and cortical areas [22]. A central role is ascribed to the dopaminergic neurons located in the ventral tegmental area and the pars compacta of the substantia nigra, both subcortical structures which, in turn, can stimulate other regions in the brain, including the nucleus accumbens, the dorsal striatum, the dorsolateral prefrontal cortex, and the ventromedial prefrontal cortex. An impairment of these neural pathways is suggested to result in reduced motivation and less ability to experience pleasure, hence promoting avolition, asociality, and anhedonia [23]. On the other hand, the pathophysiological mechanisms underlying expressive negative symptoms have received limited investigation [11], but expressive negative symptoms have been associated with impairments in neurocognitive and social cognition abilities, as well as soft neurological signs [5,11,20,21,22,23,24,25,26].

#### 3.1.1. First-Generation Antipsychotics (FGAs)

First-generation or typical antipsychotics (FGAs), including chlorpromazine, haloperidol, flupenthixol, loxapine, sulpiride, and zuclopenthixol, demonstrate a rich psycho-pharmacological profile blocking noradrenergic, cholinergic, and histaminergic receptors, in addition to blocking dopamine D2 receptors. FGAs were used not only for targeting positive symptoms, but also for the acute treatment of intense psychomotor agitation. However, an FGA-induced non-selective DA blockade results in serious adverse effects, most notably extrapyramidal symptoms (acute dystonia, parkinsonism, dyskinesia, akathisia, and tardive dyskinesia), thus placing an additional burden on the patient. Interestingly, patients with high PANSS negative scores receiving treatment with D2 dopaminergic antagonists are especially prone to developing extrapyramidal symptoms [27,28]. FGAs are still used globally as they are effective and cheap; they can be administered as long-acting injectables and, therefore, help adherence optimization at a lower cost [29].

#### 3.1.2. Second Generation Antipsychotics (SGAs)

SGAs emerged almost four decades after chlorpromazine to overcome these limitations [30]. They are characterized by a lower affinity and occupancy for the DA receptors, while 5-HT2A receptors are highly occupied, resulting in less extrapyramidal adverse effects, higher antidepressant potential, promising the alleviation of negative symptoms. Robust evidence indicated that the latter was attributed to improving secondary, not primary, negative symptoms [31]. Nevertheless, the use of SGAs spread widely due to their overall better tolerability and broader action [30,31]. When compared to placebo or FGAs, amisulpiride [15,32,33], clozapine [3,30,34,35,36], olanzapine [37,38,39,40,41], risperidone [39,42,43], and asenapine [44,45] demonstrated limited or moderate effectiveness in treating negative symptoms [46,47,48]. 

Over time, the belief that SGAs are generally better than FGAs has been questioned. In the Clinical Antipsychotic Trials of Intervention Effectiveness (CATIE) study, the effectiveness of both drug types was comparable [49]. Moreover, there were no significant differences between SGAs and FGAs regarding their effect on the severity of negative symptoms, except for olanzapine [31,38]. However, a meta-analysis based on 402 studies, including 53,463 participants, provided further insight into the efficacy and tolerability of 32 FGAs and SGAs, and concluded that olanzapine improved negative symptoms, social functioning, and depression. Nevertheless, it was one of the top drugs inducing weight gain, potentially leading to self-stigmatization and negative mood outcomes, standing in the way of being the optimal therapy for negative symptoms of schizophrenia [50]. Furthermore, a German trial of in-patients with schizophrenia found that a combination of amisulpride plus olanzapine was more effective at addressing negative symptoms compared to either drug in monotherapy [51]. Although DA plays an important role in the pathophysiology of schizophrenia, other biological processes and neurotransmitters are also critically involved [30].

#### 3.1.3. Serotonin-Dopamine Activity Modulators (SDAMs)

Aripiprazole, brexpiprazole [52,53,54], lumateperone [55], and cariprazine [56] are the four SDAMs approved by the United States Food and Drug Administration (FDA) for the treatment of schizophrenia. In contrast to previous antipsychotic medications acting as D2 receptor antagonists, SDAMs function as D2 partial agonists [57]. In other words, in the presence of excess DA, SDAMs act as D2 antagonists, whereas when DA levels are low, they stimulate the D2 receptor and act as stabilizers [58]. The hypothesis is that the partial agonist action at D2 dopamine receptors helps to reduce the excessive activity of the DA system in the striatal regions, while increasing activity in the prefrontal cortex, where DA levels are low. Each of them has distinct pharmacodynamics but they share their mechanism of action by simultaneously modulating the serotoninergic and dopaminergic systems [59,60]. 

Children and adolescents with early onset psychosis who experience more prominent negative symptoms respond better to aripiprazole than those with predominantly positive symptoms [3]. A recent meta-analysis analyzed 13 trials comparing aripiprazole to placebo, focusing on the change in PANSS negative subscales as the primary outcome and found an improvement in negative symptoms for pooled aripiprazole versus placebo [61]. In addition, an earlier meta-analysis of 168 original studies found significant effectiveness of aripiprazole on the negative symptoms [62]. Furthermore, using a network analysis approach, the effectiveness of aripiprazole at reducing negative symptoms was compared to that of other antipsychotics and placebo. The findings showed that aripiprazole was more effective than haloperidol, lurasidone, quetiapine, and cariprazine, but less effective than ziprasidone, chlorpromazine, risperidone, olanzapine, amisulpride, and clozapine [50]. Evidence for the efficacy of add-on aripiprazole in the treatment of negative symptoms was also found in treatment-resistant schizophrenia [63,64,65,66]. However, in a post hoc pooled analysis in patients with acute schizophrenia and moderate to severe negative symptoms, cariprazine demonstrated significantly greater improvement in negative symptoms compared to both placebo and aripiprazole, indicating the need for further exploration of its efficacy in treating negative symptoms [67].

In a 26-week, randomized, double-blind phase 3b study including 461 patients who had severe negative symptoms and mild positive symptoms, cariprazine monotherapy was more effective than risperidone at improving negative symptoms and functional impairment [68,69]. Consistent with this discovery, two brief phase 2 and phase 3 studies demonstrated that cariprazine is more efficacious than aripiprazole at enhancing negative symptoms in patients with an acute episode of schizophrenia [70,71]. It was thus implied that D3 receptor occupancy may be more effective than D2 receptor antagonism in addressing negative symptoms, pointing to a novel treatment approach.

Compared to several antipsychotic medications, including risperidone, olanzapine, and aripiprazole, lumateperone has a greater potency towards 5-HT2A receptors relative to D2 receptors. Thus, it is postulated that increasing the dose of lumateperone may fully activate serotonin 5-HT2A receptors before significant dopamine D2 receptor blockade occurs, reducing the risk of motor side effects both preclinically and clinically. Moreover, lumateperone appears to have positive effects on negative, positive, and cognitive symptoms without causing significant metabolic disturbances [72,73,74,75,76].

Studies on the use of brexpiprazole for the treatment of negative symptoms also showed that, compared to placebo, brexpiprazole may reduce negative symptoms [77,78,79]. 

Dopaminergic drug trials recently conducted to address negative symptoms in schizophrenia spectrum disorders are shown in Table 1.

### 3.2. The Glutamatergic System

Deficient signaling through NMDA receptors (NMDARs) has been shown to lead to morphological and structural brain modifications, and, consequently, to the development of negative symptoms. Thus, a possible therapeutic approach of negative symptoms lies in the indirect stimulation of NMDARs, with the administration of glycine receptor agonists (Table 2) [9,11]. 

Although D-Cycloserine, a partial agonist at the glycine site of NMDARs, was not effective as an adjunctive drug to dopaminergic blocking agents such as haloperidol, risperidone, or clozapine in attenuating negative symptoms [80]; the indirect modulation of NMDARs mediated by N-methyl-glycine (sarcosine), a glycine transporter (GlyT-1) inhibitor, proved advantageous as an add-on treatment [81]. Bitopterin, a glycine uptake inhibitor, has also been studied for the treatment of negative symptoms but results remain inconsistent [82,83].

The direct antagonism of NMDARs has been an attractive alternative approach for the management of negative symptoms. More specifically, the role of memantine, a non-competitive NMDAR antagonist, has been investigated [84]. Add-on memantine treatment outperformed controls with improvements of positive and negative symptoms, but not general psychopathology. In contrast, an earlier meta-analysis had found that memantine and amantadine were not superior to placebo in improving negative symptoms [85]. Despite these conflicting results, more recent studies showed the improved effectiveness of adjunctive memantine on PANSS negative symptom scores, as well as improvements in cognitive function, indicating a promising role of this medication in the treatment of negative symptoms [86,87,88,89]. Currently, the AMEND trial is ongoing, investigating the effect of add-on memantine to antipsychotic treatment as usual in reducing negative symptoms and its correlation with thalamic glutamate levels. Researchers anticipate that add-on memantine will restore regional white matter integrity and improve cognitive function [90].

Combination strategies with anticonvulsants (topiramate, lamotrigine, valproate) and antipsychotics have also been investigated for negative symptoms in schizophrenia. Topiramate is thought to exert its antiepileptic effect by inhibiting glutamatergic neurotransmission, in addition to its effects on voltage-gated Ca^2+^ and Na^+^ channels, and its antagonistic effect on the GABAergic system, among others [91]. Several meta-analyses have reported promising results on topiramate’s effectiveness for improving general and negative symptomatology when added to antipsychotic treatment, especially for clozapine augmentation [92,93,94]. Valproate and its salts are thought to modulate glutamatergic neurotransmission as well, but, in an international RCT, valproate was shown to be ineffective for negative symptoms of schizophrenia [95,96]. Interestingly, similar studies have been undertaken with lamotrigine, another glutamatergic modulator with antiepileptic and mood stabilizing properties, inhibiting the release of excitatory neurotransmitters via voltage-sensitive sodium and calcium channels, but a general recommendation for its use in the treatment of negative symptoms in schizophrenia cannot be drawn given the current evidence [97].

**Table 2:** Glutamatergic compounds recently used to address negative symptoms in schizophrenia.

Author, Year	Agent(s)	Mechanism of Action	Duration (Weeks)	No. of Subjects (N)	Negative Symptom Assessment Instrument	Findings
Kuppili et al., 2021 [80]	D-Cycloserine	Partial agonist at the glycine site of NMDARs	Study-defined endpoint (4–36 weeks) or 4 weeks (early outcome)	Seven studies (pooled N = 413) provided data for meta-analysis	PSYRATS, SANS, BPRS, PANSS	The pooled Standardized Mean Difference (SMD) for negative = symptom change scores were −0.32 (95% CI, −0.75 to 0.11). D-Cycloserine did not exhibit significant efficacy in treating negative symptoms of schizophrenia at either study-defined endpoint (4–36 weeks) or at four weeks (early outcome)
Marchi et al., 2021 [81]	N-methyl-glycine (Sarcosine)	Competitive glycine transporter −1 (GlyT-1) antagonist	4 trials 6 weeks, one 12 weeks, and one 24 weeks	Systematic review and meta-analysis (N = 6; n = 234)	Mostly PANSS negative symptoms sub-scale and SANS	No early, acute, mid-term, or long-term effect on negative symptoms
Umbricht et al., 2014 [82]	Bitopertin	Glycine reuptake inhibitor	8	323	baseline PANSS negative symptom factor score [NSFS, ≤24 vs. >24], CGI-I of Negative Symptoms (CGI-I-N)	Significantly reduced negative symptoms in patients completing a full course of eight weeks of treatment. Reduction of the PANSS NSFS score was significantly greater in the 10-mg/d and 30-mg/d groups compared to the placebo group. The mean reductions in the 60 mg group and in the placebo group were comparable. Based on the CGI-I-N scale, significantly more patients in the 10 mg group in both analysis populations were rated much or very much improved compared to the placebo group.
Bugarski-Kirola et al., 2017 [83]	Bitopertin	Glycine reuptake inhibitor	24	DayLyte [WN25309] N = 605 and FlashLyte [NN25310] N = 594	PANSS Negative Symptom Factor Score (NSFS)	This study did not support prior results showing beneficial effect of adjunctive bitopertin in negative symptoms of schizophrenia.
Kishi and Iwata, 2013 [85]	Memantine Amantadine Add-on to TAU	Memantine is non-competitive antagonist for NMDARs, serotonin-3 receptors, and nicotinic acetylcholine receptors (nAChR), including α-7, and it is also a dopamine D2 receptor agonist. Amantadine is a NMDAR antagonist and shares a nicotinic acetylcholine receptor (nAChR) alpha-7 antagonist effect with memantine.	Meta-analysis	Memantine (N = 3, n = 186) Amantadine (N = 5, n = 220)	PANSS negative subscale	NMDA receptor antagonists (NMDAR-ANTs) as adjunctive therapy were not superior to placebo in negative symptoms (SMD = −0.69, CI = −1.65, 0.27, *p* = 0.16, N = 4, n = 205)
Rezaei et al., 2013 [89]	Memantine vs. Placebo Add-on to Risperidone 6mg/day	NMDAR antagonist	8	40	Extrapyramidal Symptom Rating Scale (ESRS)	Patients in the memantine group showed a significantly greater improvement on negative subscale compared to the placebo group at end point (*p* < 0.001).
Matsuda et al., 2013 [88]	Memantine Amantadine Add-on to TAU	NMDAR antagonists	Meta-analysis (2–24 weeks)	Memantine (N = 4, n = 222) Amantadine (N = 5, n = 220)	PANSS-negative	Memantine: SMD = −1.08 (−2.21, 0.04), *p* = 0.06
Kishi et al., 2017 [87]	Memantine Add-on to TAU	Blockade of current flow through channels of N-methyl-d-aspartate (NMDA) receptors. Modulation of NMDA receptor activity can increase or decrease excitability of neuronal circuits.	Meta-analysis (6–26 weeks)	N = 8, n = 448	PANSS -negative, BPRS-negative	Memantine add-on treatment was superior to placebo for ameliorating negative symptoms (SMD = −0.96, 95% CIs = −1.64 to −0.27, *p* = 0.006, I^2^ = 88%; N = 7, n = 367)
Veerman et al., 2016 [86]	Add-on Memantine to refractory patients with schizophrenia on Clozapine	NMDAR antagonist	12	52 Double-blind adjunctive treatment with memantine (n = 26) or placebo (n = 26)	PANSS	When compared with placebo, memantine improved PANSS negative subscale score (effect size = 0.29)
Zheng et al., 2019 [84]	Memantine vs. Placebo adjunct to TAU	Non-competitive NMDA receptor antagonist		N = 15 RCTs (n = 988): schizophrenia (N = 9, n = 512), bipolar disorder (N = 3, n = 319), and MDD (N = 3, n = 157)	PANSS -negative, BPRS-negative	Memantine outperformed the comparator regarding negative symptoms with an SMD of −0.71 (95% CI: −1.09, −0.33; I^2^ = 74%, *p* = 0.0003) in schizophrenia
Zheng et al., 2016 [94]	Topiramate	Glutamate modulation through kainate/AMPA receptor antagonism, state-dependent blockage of voltage-activated Na^+^ channels and enhancement of GABA activity at GABA_A_ receptors.	11.8 ± 5.6	Random-effects meta-analysis of 16 RCTs (n = 934) of topiramate cotreatment with antipsychotics vs. placebo/ongoing antipsychotic treatment in schizophrenia-spectrum disorders	PANSS -negative	Topiramate outperformed the comparator regarding change/endpoint of negative symptoms (SMD: −0.58, 95% CI: −0.87, −0.29, *p* < 0.0001)
Veerman et al., 2014 [93]	Lamotrigine	An antagonist of postsynaptic voltage-sensitive sodium channels, decreasing presynaptic release of glutamate	10–24	Meta-analysis of N = 6 studies, including n = 185 participants with refractory schizophrenia on clozapine	PANSS-N, SANS	Lamotrigine did not differ from placebo with regard to reducing negative symptoms (ES = 0.367, *p* = 0.163; 95 %CI = −0.148 to 0.883).
Ibrahim et al., 2019 [96]	Valproate	Increases GABA levels by increasing succinic semialdehyde, an endogenous inhibitor of GABA-transaminase (GABA-T). Valproate also exerts a direct effect on excitable membranes and attenuates the NMDA receptor channel.	20	RCT of Valproate or placebo as adjuncts to risperidone treatment in patients with early course schizophrenia (N = 109)	PANSS	No significant differences between Valproate- and placebo-treated groups with respect to changes in negative symptom scores at the beginning and end of the study.

### 3.3. The Inflammatory Pathway

Evidence suggests that inflammatory processes may play a seminal role in the pathophysiology of schizophrenia. Notably, negative symptoms have been associated with altered cytokine levels, namely elevated TNF-α, Il-1β, Il-8, IFN-γ, Il-4, and TGF-β, and decreased Il-10 in chronic and drug-naive FEP, respectively [98]. Therefore, anti-inflammatory agents have been postulated as valuable therapeutic agents for schizophrenia (Table 3). Aspirin, estrogens, minocycline, and N-acetylcysteine (NAC) were found to be effective, confirming previous positive RCTs [99,100]. Interestingly, an earlier 12-week RCT including 82 patients with schizophrenia from Iran had shown significant improvement in negative symptoms when NAC was added to antipsychotic treatment [101]. A meta-analysis from Australia found that NAC may be a valuable adjunct to standard treatment for improving negative symptoms and the cognitive domain of working memory in patients with FEP. Treatment effects were observed at or later than 24 weeks, suggesting that longer interventions may be required for effectiveness in adding NAC to treatment as usual [102]. Nevertheless, a large meta-analysis on the efficacy of anti-inflammatory drugs demonstrated no significant effects of pregnenolone, statins, bexarotene, celecoxib, davunetide, dextromethorphan, fatty acids, or varenicline [103]. Moreover, a most recent meta-analysis evaluating the efficacy of NAC as an augmentation strategy could not find a time-dependent or any other beneficial effect of NAC against placebo [104]. Results from several other studies remain conflicting [105,106,107,108,109]. 

Abnormal lipid metabolism, such as reduced polyunsaturated essential fatty acids (PUFAs), particularly omega-3 PUFAs, is also thought to be involved in the pathophysiology of schizophrenia. Depletion of omega-3 PUFAs in the brain has been linked to oxidative damage [110]. However, further meta-analyses report contradictory findings, providing no established confirmation of whether dietary supplementation with omega-3 PUFAs has any effect on schizophrenia symptoms [111].

A review of 41 studies on anti-inflammatory agents, including amino acids, anti-inflammatory drugs, and hormonal therapies, showed that NAC and polyunsaturated fatty acids (PUFAs) had significant effects in reducing negative symptoms and may be recommended for clinical practice. Additional research is, however, needed for treatment individualization according to the phase of the illness, as inflammatory markers appear to differ between acute episodes and stable periods of schizophrenia [98].

**Table 3:** Anti-inflammatory compounds recently used to address negative symptoms in schizophrenia.

Author, Year	Agent(s)	Mechanism of Action	Duration (Weeks)	No. of Subjects (N)	Negative Symptom Instrument	Findings
Çakici et al., 2019 [103]	Aspirin, bexarotene, celecoxib, davunetide, dextromethorphan, estrogens, fatty acids, melatonin, minocycline, Ν- acetylcysteine (NAC), pioglitazone, piracetam, pregnenolone, statins, varenicline, and withania somnifera extract	Antioxidant and anti-inflammatory actions. Inhibition of COX-1, COX-2, CRP and proinflammatory cytokines, such as IL-1β, IL-6, TNF-α, IFN-γ, NF-κβ.	N/A	Minocycline 8–26 weeks NAC 8–52 weeks Estrogen 2–12 weeks	PANSS	Augmentation therapy with minocycline [N = 10 RCTs with 11 treatment arms, n = 946 (ES: 0.50; 95% CI, 0.17 to 0.84; *p* = 0.003; I^2^ = 82%)], NAC [N = 5, n = 442 (ES: 0.75; 95% CI, 0.19 to 1.32; *p* = 0.009; I^2^ = 88%)], and estrogen [N = 11 RCTs with 12 treatment arms, n = 723 (ES: 0.45; 95% CI, 0.13 to 0.77; *p* = 0.006; I^2^ = 73%)] showed positive results for improving negative symptoms. Melatonin, pioglitazone, piracetam, pregnenolone, and withania somnifera extract (WSE) were investigated in single studies only and seem to have beneficial effects on negative symptoms.
Berk et al., 2008 [99]	NAC (1 g orally twice daily) as an add-on to maintenance medication for the treatment of chronic schizophrenia		24	140 randomized, 82 completed	PANSS negative	Intent-to-treat analysis revealed that subjects treated with NAC improved more than placebo-treated subjects over the study period in PANSS negative [mean difference −1.83 (95% confidence interval: −3.33, −0.32), *p* = 0.018]
Breier et al., 2018 [100]	NAC (3600 mg/day)		52	N = 60 early phase schizophrenia spectrum disorders	PANSS negative	NAC significantly improved (time × group) PANSS negative (F = 5.1, *p* = 0.024) symptom scores
Neill et al., 2022 [107]	Adjunctive NAC 2 mg daily in clozapine patients with enduring psychotic symptoms		8, 24, 52	84	PANSS -negative subscale	NAC did not significantly improve negative symptoms (*p* = 0.62) at any time point over a one-year period of treatment in clozapine resistant schizophrenia
Farokhnia et al., 2013 [108]	NAC (up to 2 g/d) or placebo, in addition to risperidone (up to 6 mg/d)		8	42	PANSS -negative subscale	NAC-treated patients showed significantly greater improvement in the PANSS negative subscale (*p* < 0.001) scores than that in the placebo group
Sinichi et al., 2023 [109]	400 mg dose of Pentoxifylline or placebo bd		8	52	PANSS -negative subscale	No significant difference was observed in negative symptoms
Yolland et al., 2020 [102]	N-acetylcysteine as an adjunct treatment for schizophrenia		24	Meta-analysis N = 7, n = 440	PANSS -negative subscale	No significant difference between NAC and placebo groups in negative symptoms at the ≤8-week time point. At the ≥24-week time point, NAC did significantly improve negative symptoms in comparison to placebo, with a moderate effect size (SMD = −0.41, *p* = 0.006). Combining data from the final end-point of each study, for PANSS negative, a statistically significant large overall effect was observed (SMD = −0.72, *p* = 0.003).
Sepehrmanesh et al., 2018 [101]	Adjunctive NAC 1200 mg to TAU		12	84	PANSS negative subscale	NAC-treated patients showed significant improvement in the negative (F = 0.20, df = 1) PANSS subscale
Zhang et al., 2024 [104]	N-acetylcysteine (NAC)	NAC acts as (i) a reductant of disulfide bonds, (ii) a scavenger of reactive oxygen species, and/or (iii) a precursor for glutathione (GSH) biosynthesis. Inhibition of proinflammatory cytokines IL-1β, IL-6, TNF-α.	Grouped at ≤24 weeks and >24 weeks	N = 8, n = 594	PANSS	No difference was found in score changes of PANSS negative scale scores between the NAC group and placebo group in both time points (≤24 weeks and >24 weeks)

### 3.4. The Serotonin System

The involvement of the serotonergic system in the pathophysiology of schizophrenia has been suggested by the effectiveness of SGAs on negative symptoms. Moreover, secondary negative symptoms are frequently emerging from depressive symptoms, and both improve with antidepressant treatment. However, whether symptomatic improvements in schizophrenia are due to the improvement of negative or depressive symptoms remains to be determined. A meta-analysis of 42 studies (n = 1934) showed that add-on antidepressants had small-to-medium effectiveness for decreasing negative symptoms, and negative symptom improvement varied across antidepressant compounds and appeared specific to FGA augmentation [112]. The most potent add-on antidepressants were mirtazapine and fluoxetine. They proved to be more effective than antipsychotic monotherapy on negative symptomatology. Recent clinical trials further support negative symptom improvement by adding fluvoxamine to risperidone [113], duloxetine to risperidone [114], and the addition of escitalopram to antipsychotics [115]. Similarly, Salazar de Pablo et al. (2023) showed that antidepressants are beneficial for adults with early onset psychosis and negative symptoms, but with a small effect size [3].

A different medication class targeting serotonin receptors, specifically the 5-HT3 receptor, has been recently explored in schizophrenia [116]. In addition to its established role as an anti-emetic agent, a small clinical trial from Iran [117] and a meta-analysis of five RCTs (n = 304) [118] claim that combining ondansetron, a 5-HT3 antagonist, with antipsychotics leads to significant negative symptom reduction and improvement in overall psychopathology.

Roluperidone (MIN-101), a novel compound that acts as a 5HT2A, sigma 2 (σ2) receptor antagonist has been reported as promising in the attenuation of negative symptoms of schizophrenia. Davidson et al. (2017) first demonstrated its efficacy and tolerability in a phase 2b clinical trial which included 234 patients with acute negative symptoms and a relatively stable course of positive symptoms [119]. In this study, MIN-101 exhibited efficacy compared to placebo at both dosages of 32 mg/day and 64 mg/day. Importantly, improvement in negative symptoms was not attributed to improvements in depressive or extrapyramidal symptoms. The validity of these results, and, by extension, the effectiveness of roluperidone, was replicated by a re-analysis of the same data, with negative symptoms as the outcome of reduced expression and reduced experience. In both treatment groups, at different time points, the drug proved to be superior to placebo equally in both domains, further supporting its beneficial effect in people with schizophrenia [120]. Finally, Rabinowitz and his colleagues (2019) [121] suggested a beneficial effect of roluperidone in personal and social adjustment in patients with stable positive and severe negative symptoms [122].

Lastly, pimavanserin, a selective 5-HT2A inverse agonist and antagonist, appeared to be a promising option as it reduced negative symptoms in stable patients according to a phase 2 randomized, placebo-controlled trial, but further investigation is needed to determine whether the effect was clinically significant [122].

Recent clinical trials of serotonergic compounds in the fight against negative symptoms are tabulated in Table 4.

### 3.5. The Cholinergic System 

Schizophrenia is associated with significant changes in the cholinergic system [123]. Anticholinergic drugs have been used to alleviate extrapyramidal symptoms, but they come with adverse side-effects and reduced efficacy with prolonged use. Studies have explored treatments that target acetylcholinesterase (AChE), the acetylcholine degradation enzyme. A meta-analysis revealed that adding AChE inhibitors such as donepezil, galantamine, or rivastigmine to antipsychotics can improve negative symptoms in patients with schizophrenia [124]. Moreover, cytidine 5′-diphosphocholine (Citicoline; CDP-choline) a selective α7 nicotinic acetylcholine-receptor (nAChR) agonist with neuroprotective properties, significantly improves primary negative symptoms when added to risperidone [125]. The combination of CDP-choline and galantamine has been reported to show greater improvement in negative symptoms in individuals with higher PANSS-baseline scores [126]. The potential benefits of combining galantamine with memantine as an antioxidant have also been reported [127]. Table 5 summarizes two most recent clinical trials involving cholinergic drugs for the treatment of negative symptoms in schizophrenia.

### 3.6. The Noradrenergic System

Another neurotransmitter system suggested to have an impact on both positive and negative symptomatology in schizophrenia is the noradrenergic system, with noradrenaline being crucially involved in cognition and basic information processes. Via projections from the locus coeruleus to the prefrontal cortex, noradrenaline acts on α1-receptors, associated with stress and cognitive dysfunction, and on postsynaptic α2-receptors associated with enhanced cognition [128]. One recent double-blind, randomized controlled trial investigating the effect of clonidine, a selective α2-agonist, in 32 chronic schizophrenia patients found that 50 μg clonidine daily over a course of six weeks significantly reduced negative symptom severity (Table 6) [129]. Additional studies with larger groups of patients are needed to confirm its promising therapeutic effect.

### 3.7. Psychostimulants

Psychostimulants have shown potential as an adjunctive therapy for treating negative and cognitive symptoms of schizophrenia. A retrospective review from 2014 to 2019 examined 77 outpatients receiving psychostimulants. The majority showed improvement in their cognitive symptoms, although one third of the patients developed psychosis. Among other factors, comorbid attention deficit hyperkinetic disorder (ADHD) was associated with a greater probability of response. However, higher stimulant doses increased the probability of the emergence of psychosis [130]. Moreover, a multicenter, open-label, double-blind, placebo-controlled randomized-withdrawal study was carried out recruiting outpatients with schizophrenia at 28 US sites from September 2009 to January 2011. When lisdexamfetamine dimesylate, a pro-drug of D-Amphetamine, was administered as an adjunctive therapy to antipsychotics, a significant reduction in negative symptom severity was observed [131]. These results have not been replicated by others, however [132]. More recently, a meta-analysis of randomized controlled trials evaluating the effect of dopamine partial agonists and pro-dopaminergic drugs revealed no overall improvement of psychopathology for pooled pro-dopaminergic drugs. However, in a subgroup-analysis for negative symptom severity, ar/modafinil was found to be superior to placebo [61]. 

### 3.8. Hormones 

Schizophrenia exhibits sex differences both in prevalence and course. Men present a worse clinical course than women, with more severe social, cognitive, and neuro-behavioral deficits. On the other hand, only women have a second age peak for schizophrenia onset, which usually coincides with the premenopausal stage at the start of hormonal decline, suggesting that estrogen may have a protective role in the pathophysiology of schizophrenia [133]. Indeed, several clinical trials have suggested the beneficial effect of estrogens, such as raloxifene, a selective estrogen receptor modulator (SERM), that attenuated symptoms when used in combination with antipsychotic medication. Patients who received estrogen add-on therapy showed improved functional and clinical outcomes, and required lower antipsychotic dosages [134]. Nevertheless, in a recent clinical trial, raloxifene did not improve symptoms in patients with schizophrenia spectrum disorders. However, it showed beneficial effects on negative symptoms and working memory in women [135]. 

Studies have also explored oxytocin, a neuropeptide known to mediate social behavior, as a potentially valuable compound that can help patients with schizophrenia and intense negative symptoms. It has been shown that endogenous oxytocin levels are inversely correlated with negative symptomatology; low levels have been related to severe negative symptoms such as social withdrawal, isolation, and flattened effect [136]. Exogenously supplied oxytocin combined with clozapine has been found to reduce negative and conserve moderate positive symptoms (as assessed by the PANSS), and improve social functioning in a small group of adults with treatment-resistant schizophrenia [137]. However, a recently published meta-analysis of randomized, double-blind, placebo-controlled trials did not report an overall beneficial effect of administered oxytocin [138]. Additional studies with larger groups of patients are needed to confirm its potential therapeutic effect. 

Table 7 summarizes recent trials using hormones to address negative symptoms in schizophrenia.

## 4. Discussion

Diagnosing and treating negative symptoms remains challenging, despite multi-level approaches to their management. Emerging evidence from different research teams around the globe has provided valuable insights into defining and distinguishing negative symptoms and addressing them with several potentially therapeutic pharmacological agents [48]. Results are, however, largely inconsistent. 

Several treatment guidelines provide recommendations for the treatment of negative symptoms of schizophrenia, but the level of evidence for the recommendations and the anticipated therapeutic effects vary greatly. According to the British Association for Psychopharmacology (BAP), available antipsychotic medication has limited effectiveness for negative symptoms. Amisulpride is approved for treating schizophrenia with prominent negative symptoms, including in patients with predominant negative symptoms. Despite the lack of convincing evidence, certain patients may benefit from pharmacological treatments such as antidepressants, antipsychotics, or glutamatergic drugs. BAP recommended using CAINS or BNSS to monitor response to treatment and whether or not to continue with treatment according to clinical improvement [140]. The World Federation of Societies of Biological Psychiatry (WFSBP) Task Force on Treatment Guidelines for Schizophrenia (2012) recommended SGAs for the treatment of negative symptoms, with amisulpride and olanzapine being classified as effective at Category of Evidence A, Recommendation grade 1, for both primary and secondary negative symptoms [141]. The Scottish Schizophrenia guidelines recommend (Category B) antipsychotic augmentation with an antidepressant, lamotrigine or sulpiride, for persistent negative symptoms [142]. The CINP Guidelines [143] and the Polish guidelines [144] agreed on the effectiveness of amisulpride, but further recommend the use of low doses to avoid the emergence of secondary negative symptoms, extra-pyramidal, and metabolic side-effects. The Polish guidelines recommended olanzapine, aripiprazole, quetiapine, lurasidone, paliperidone, risperidone, ziprasidone, and sertindole as effective treatments for negative symptoms, as well as switching to clozapine in refractory cases. The German guidelines also recommend amisulpride and olanzapine at strength of recommendation B and suggest the addition of an antidepressant in cases where antipsychotic monotherapy has proved ineffective [145]. Moreover, the French guidelines on the use of long-acting antipsychotics state that SGA LAIs should be used as a second line of treatment for negative symptoms in schizophrenia [146]. According to the Japanese Expert Consensus guidelines, oral aripiprazole was the only first choice for schizophrenia with predominant negative symptoms, and aripiprazole LAI, followed by paliperidone palmitate, were considered to be first-line treatment for negative symptoms [147]. Furthermore, antipsychotic effects on negative symptoms do not differ to a clinically significant extent between clozapine and other antipsychotic medications [140]. The European Psychiatric Association (EPA) guidance on the treatment of negative symptoms of schizophrenia recommends (i) an antipsychotic with antidepressant properties or the addition of an antidepressant for patients with negative symptoms and depression, (ii) optimization of the antipsychotic treatment for negative secondary to positive symptoms, and (iii) clozapine for negative symptoms secondary to treatment-resistant positive symptoms [48]. Moreover, they recommend a switch to an SGA if patients with negative symptoms are treated with a FGA, in line with the Spanish guidelines [148].

A more recently published network meta-analysis of 45 compounds added to risperidone, mixed antipsychotics, or clozapine was conducted to determine the efficacy of augmentation drugs for the treatment of schizophrenia. Most notably, tropisetron, memantine, and minocycline were found to address negative symptoms among patients treated with risperidone effectively. Moreover, memantine demonstrated efficacy for negative symptoms in studies with mixed antipsychotics, and, in clozapine augmentation trials, duloxetine was the only drug effectively treating negative symptoms. However, several important drugs did not appear in the results, possibly due to a lack of reliable data. Nevertheless, the findings indicate novel ways to treat schizophrenia that should be included in future guidelines, pending further validation [149]. 

There is a significant unmet need for treating the primary negative symptoms of schizophrenia. The development of new compounds and the re-evaluation of existing agents is of great importance. Additional trials should be conducted with these agents, considering factors such as trial duration, exclusion of patients with secondary negative symptoms, use of active comparators, and assessment using specialized scales. Most importantly, patient-centered outcome measures that assess real-world relevance, such as functioning and quality of life, should also be collected and taken into consideration [4]. Large-scale clinical trials and well-established meta-analyses will eventually shed light on the optimal treatment of negative symptoms.

Moreover, biomarkers that could be used to define patients expressing negative symptoms or predict their treatment response are desperately needed to advance our understanding of the mechanisms that underlie the highly complex pathophysiology of negative symptoms in schizophrenia and other closely related psychiatric diseases.

Furthermore, besides pharmacological interventions, non-pharmacological treatments have been found to improve symptoms of schizophrenia [150]. Non-invasive clinical treatments, such as electroconvulsive therapy (ECT) and repeated transcranial magnetic stimulation (rTMS), in particular of the left prefrontal cortex, have been shown to decrease negative symptomatology in schizophrenia patients [151,152,153]. Furthermore, intermittent theta burst stimulation (iTBS) can effectively improve negative symptoms and social cognition of individuals with schizophrenia and ameliorate their functional recovery [154]. 

Another potent approach of immense importance is psychological therapy. Psychological interventions, including cognitive behavioral therapy (CBT), social skills training, psychoeducation, cognitive remediation (CR), and integrated psychological therapy (IPT), can provide significant benefits for negative symptoms of patients with schizophrenia, but more studies are needed [92,155,156]. Additionally, life skills training, supported housing and employment, lifestyle modifications such as a healthy diet and physical activity, and music and art therapies, may also benefit schizophrenia patients with exercise-based therapy, significantly reducing negative symptoms [157,158,159]. These non-pharmacological interventions, however, are beyond the scope of this review.

## 5. Conclusions

In conclusion, this selective review provides an overview of both novel and established pharmacological strategies used to address negative symptoms in schizophrenia. However, none of the pharmacological interventions described here is approved by the Food and Drug Administration, the European Medicines Agency, or other major regulatory agencies for the treatment of negative symptoms at present, and the treatment of negative symptoms remains largely an unmet need. Scientific knowledge seems to be currently insufficient to explain clearly the pathophysiology of negative symptoms within the schizophrenia spectrum. Nevertheless, all available interventions could be considered with caution on an individualized basis. Several treatment guidelines offer recommendations, but the evidence for the recommendations and the anticipated therapeutic effects vary greatly. Most of the literature supporting pharmacologic treatments needs more rigor as clinical studies have small sample sizes and marked heterogeneity. High-quality research will advance our understanding of key aspects of these debilitating and stigmatizing symptoms of schizophrenia, leading to much-needed improvements in the quality of life of patients with schizophrenia, as well as their carers, clinicians, and society as a whole.

## Figures and Tables

**Table 1:** Dopaminergic compounds recently used to address negative symptoms in schizophrenia.

Author, Year	Agent(s)	Mechanism of Action	Duration (Weeks)	No. of Subjects (N)	Negative Symptom Instrument	Findings
Liang and Yu, 2017 (post hoc) [33]	Amisulpride	Partial agonist D2/D3, antagonist 5-HT7	8	26	PANSS negative, CGI-S	Amisulpride treatment not only reduces NS but also provides a meaningful clinical benefit
Miller et al., 1994 [34]	Clozapine	D2, 5-HT2A antagonist	6	29	SANS	31% improvement in NS, 32% improvement in psychotic symptoms, 35% improvement in disorganization
Butler et al., 2022 [35]	Clozapine	D2, 5-HT2A antagonist	At discharge, not further specified	88	PANSS	Improvement of NS
Novick et al., 2017 (post hoc) [38]	Olanzapine Other SGAs FGAs	D2/D3 antagonism, D2 partial agonism, 5-HT2A antagonism, 5-HT1A partial agonism, 5-HT2C antagonism, M1 antagonism, M3 antagonism, M1 agonism, H1 antagonism, α1/2 antagonism	156	3712	CGI-SCH	Olanzapine superior over other SGAs (risperidone, quetiapine, amisulpride, clozapine); adjusted mean change in the CGI-SCH negative symptoms scores during follow-up was greater for olanzapine-treated patients by 0.220 (*p* < 0.001) (vs. other atypicals) and by 0.453 (*p* < 0.001) (vs. typicals)
Takekita et al., 2022 [45]	Asenapine	D2, 5HT2A α_2_ adrenoceptor antagonist	6	529	PANSS DIEPSS	Improvement of NS; no significant difference from the placebo group was observed in DIEPSS change at the final evaluation
Schmidt-Kraepelin et al., 2022 (DB/RCT) [51]	Olanzapine + Amisulpride	D1-D5/5-HT2A/5-HT2c/5-HT3/5-HT6/α-1/H1/M1-5 antagonism + partial agonist D2/D3, antagonist 5-HT7	16	328	PANSS total	Olanzapine + Amisulpride combination efficient for NS and superior over either monotherapy.
Earley et al., 2019 [67]	Cariprazine vs. Placebo and Cariprazine vs. Aripiprazole (post hoc pooled analysis)	D2/D3, 5-HT1A partial agonist	6	Cariprazine [n = 160] vs. Placebo [n = 79] and Cariprazine [n = 160] vs. Aripiprazole [n = 44]	Positive and negative syndrome scale factor score for negative symptoms (PANSS-FSNS)	Cariprazine was associated with significantly greater improvement in moderate/severe negative symptoms compared to placebo and aripiprazole.
Fleischhacker et al., 2019 [68]; Németh et al., 2017 [69]	Cariprazine vs. Risperidone	D2/D3, 5-HT1A partial agonist	26	Cariprazine [n = 227] vs. Risperidone [n = 229]	PANSS-FSNS >24	Both improved NS, superiority of Cariprazine [−8.63 (0.32)] vs. Risperidone [−7.16 (0.34)], mean difference [−1.48 (−2.38 to −0.57), *p* = 0.0015] in reducing NS
Lieberman et al., 2016 [75]	Lumateperone	D2/5-HT2A receptors	4	Lumateperone 60 mg [n = 60] Lumateperone 120 mg [n = 64]	PANSS CDSS	Lumateperone 60 mg improved negative symptoms and depressive symptoms in patients with depression at baseline. However, Lumateperone 120 mg did not differ from placebo
Correll et al., 2020 [76]	Lumateperone	D2/5-HT2A receptors	4	Lumateperone 42 mg [n = 150] Lumateperone 28 mg [n = 150]	PANSS	Twenty-eight (28) mg lumateperone did not improve PANSS negative subscores. However, 42 mg lumateperone improved positive symptoms and negative symptoms [Least squares mean change (LSMC) −4.2, multiplicity-adjusted *p* = 0.04]
Osugo et al., 2022 [61]	Prodopaminergic drugs, including DA partial agonists (aripiprazole, brexpiprazole, cariprazine, bifeprunox, preclamol, and brilaroxazine) and prodopaminergic treatments (modafinil, armodafilnil, dexamphetamine, lisdexamphetamine, L-DOPA, bromocriptine, apomorphine/N-propylnorapomorphine, methylphenidate, pramipexole, tolcapone, and the ergot dopamine agonist CF-25–397)	A systematic review and meta-analysis to investigate the effect of all medications which increase dopamine signalling on symptoms of schizophrenia, using all available data from placebo controlled, double-blind RCTs	N/A	55 articles (describing 59 trials): 30 trials (N = 9967) of dopamine partial agonists (aripiprazole im and po, brexpiprazole, cariprazine, bifeprunox, preclamol, and brilaroxazine) and 19 trials (N = 860) of prodopaminergic treatments (modafinil, armodafilnil, dexamphetamine, lisdexamphetamine, L-DOPA, methylphenidate, and tolcapone)	-	Dopamine partial agonists (aripiprazole, brexpiprazole, cariprazine, bifeprunox, preclamol, and brilaroxazine) were significantly superior to placebo in the treatment of negative symptoms (SMD = −0.29, 95% CI −0.34; −0.24, *p* = 2.2 × 10^−31^). Pooled ar/modafinil was found to be significantly superior to placebo in the treatment of negative symptoms (SMD = −0.34, *p* = 0.037, 95% CI = −0.66; −0.02, *p* = 0.037).

**Table 4:** Serotonergic compounds recently used to address negative symptoms in schizophrenia.

Author, Year	Agent(s)	Mechanism of Action	Duration (Weeks)	No. of Subjects (N)	Negative Symptom Assessment Instrument	Findings
Galling et al., 2018 [112]	Antidepressants	SSRI, SARI SNRI, NaSSA, NRI, TCA, MAO-inhibition	10.1 ± 8.1 weeks	N = 32, n = 1348	PANSS negative subscale, BPRS negative subscale, SANS	Antidepressant augmentation was superior to placebo for negative symptom reduction (SMD = −0.25, 95% CI = −0.44 to −0.06, *p* = 0.010). Superiority regarding negative symptoms was confirmed in studies augmenting first-generation antipsychotics (FGAs) (SMD = −0.42, 95% CI = −0.77, −0.07, *p* = 0.019), but not second-generation antipsychotics (*p* = 0.144). Uniquely, superiority in total symptom reduction by NaSSAs (SMD = −0.71, 95% CI = −1.21, −0.20, *p* = 0.006) was not driven by negative (*p* = 0.438), but by positive symptom reduction (SMD = −0.43, 95% CI = −0.77, −0.09, *p* = 0.012).
Javadi et al., 2018 [113]	Fluvoxamine + Risperidone + Biperiden vs. Risperidone + Biperiden	Selective reuptake inhibition of 5-HT, σ_1_ agonism	10	68	SANS	The difference between the groups in terms of SANS scores was significant in the specified intervals (8 and 10 weeks); SANS (F = 6.36, *p* = 0.004).
Nikbakhat et al., 2016 [114]	Duloxetine (60 mg/day) or placebo add-on to risperidone (up to 6 mg/day)	Selective Reuptake Inhibition of 5-HT and NA leading to DA increase in PFC	8	64	PANSS negative	Compared to the placebo group, the duloxetine group showed significantly higher improvement in negative symptoms (*p* < 0.001).
Ding et al., 2018 [115]	Escitalopram	Selective reuptake inhibition of 5-HT, decreases IL-6 and CRP levels	8	62	PANSS negative	Reductions in PANSS negative subscore were more important in escitalopram treated patients than in the placebo group (*p* < 0.05).
Samadi et al., 2017 [117]	Ondansetron; risperidone either combined with a fixed dose (4–8 mg/d) of ondansetron (n = 18) or with a placebo (n = 20)	5-HT3 antagonist	12	38	PANSS negative subscale	Ondansetron plus risperidone was associated with a significantly larger improvement in the PANSS subscale for negative symptoms than was risperidone plus placebo (*p* < 0.001).
Zheng et al., 2019 [118]	Ondansetron (4–8 mg/day)	A potent 5-HT3 receptor antagonist		N = 5 RCTs (n = 304)	PANSS negative subscale	Adjunctive ondansetron outperformed placebo in the reduction of the negative [4 RCTs, n = 209; SMD: -0.96 (95%CI: −1.71, −0.22), *p* = 0.01, I^2^ = 80%] symptom scores
Davidson et al., 2017 [119]	Randomly assigned to receive placebo or 32 mg/day or 64 mg/day of MIN-101 for 12 weeks	5HT_2A_, Sigma_2_ antagonism	12	244 symptomatically stable for at least three months with scores of ≥20 on the negative subscale of PANSS	PANSS negative factor, SANS	A statistically significant difference in PANSS negative factor score was observed, with lower scores for the MIN-101 32 mg/day and 64 mg/day groups compared with the placebo group (effect sizes, d = 0.45 and d = 0.57, respectively)
Harvey et al., 2020 [120]	Daily monotherapy with Roluperidone 32 mg, Roluperidone 64 mg, or placebo in a 1:1:1 ratio.	5HT_2A_, Sigma_2_ antagonism	12	244 symptomatically stable patients with schizophrenia with baseline scores ≥20 on the NS subscale of the PANSS	PANSS negative subscale	Both doses of roluperidone were superior to placebo on both domains: Reduced Experience (*p* ≤ 0.006 for the 32 mg; *p* ≤ 0.001 for the 64 mg) with persistent superiority from Week 2 for the 64 mg dose and Week 8 for the 32 mg dose; Reduced Expression (*p* ≤ 0.003 for 32 mg; *p* ≤ 0.001 for 64 mg) with similar persistence.
Bugarski-Kirola et al., 2022 [122]	Pimavanserin add- on or placebo to TAU	A selective 5-HT_2A_ inverse agonist and antagonist	26	403	16-item Negative Symptom Assessment (NSA-16)	The change in total NSA-16 score from baseline to week 26 was significantly improved with pimavanserin (least squares mean −10.4 [SE 0.67]) versus placebo (least squares mean −8.5 [0.67]; *p* = 0.043; effect size: 0.211).

**Table 5:** Cholinergic trials for the treatment of negative symptoms in schizophrenia.

Author, Year	Agent(s)	Mechanism of Action	Duration (Weeks)	No. of Subjects (N)	Negative Symptom Assessment Instrument	Findings
Ghajar et al., 2018 [125]	Cytidine 5′-diphosphocholine (CDP-choline or citicoline) 2500 mg/day or placebo in addition to risperidone	Selective α7 nAChR agonist	8	66	PANSS ESRS	The citicoline group demonstrated significantly greater improvement in negative scores, F(1.840, 118.360) = 8.383, *p* = 0.001.
Choueiry et al., 2023 [126]	Combination CDP-choline 500 mg and galantamine 16 mg	Selective α7 nAChR agonist (α7 nAChR modulate glutamatergic and NMDA receptor signaling in the hippocampal CA1 region) and Acetylcholinesterase inhibitor (nicotinic allosteric modulator)		24 stable patients with schizophrenia stratified by baseline MMN responses into low, medium, and high baseline auditory deviance detection subgroups	Speech MMN reflecting deficits in early auditory prediction-error processes (i.e., in pre-attentive sensory processing)	CDP-choline/galantamine significantly increased MMN amplitudes to frequency, duration, and vowel speech deviants in low group individuals. Individuals with higher PANSS negative scores expressed the greatest MMN amplitude improvement following CDP-choline/galantamine.

**Table 6:** Clonidine RCT on negative symptoms in schizophrenia.

Author, Year	Agent(s)	Mechanism of Action	Duration (Weeks)	No. of Subjects (N)	Negative Symptom Assessment Instrument	Findings
Kruiper et al., 2023 [129]	Clonidine	Selective α_2_-agonist	6	53	PANSS negative scale	Only patients treated with clonidine showed significantly reduced PANSS negative

**Table 7:** Hormones recently used to address negative symptoms in schizophrenia.

Author, Year	Agent(s)	Mechanism of Action	Duration (Weeks)	No. of Subjects (N)	Negative Symptom Assessment Instrument	Findings
Sabe et al., 2021 [77]	Oxytocin (fixed doses range: 40, 48, 80 IU/d)	Secreted by the posterior pituitary gland, modulates several aspects of behavior through regulation of its central dopaminergic action.	3–16 (mean = 7.3 weeks)	N = 9, n = 308	SANS PANSS BPRS	IN oxytocin was not superior to placebo with respect to the mean change in negative symptoms (SMD = −0.26; 95% CI = −0.61, 0.09; *p* = 0.14)
Brand et al., 2023 [135]	Raloxifene add-on or placebo to TAU	Selective estrogen receptor modulator.	12	102	PANSS negative scale	In women (28% of participants), raloxifene had beneficial effects on negative symptoms at week 6 (LSM −2.92; adjusted *p* = 0.020) and week 12 (LSM −3.12; adjusted *p* = 0.030)
Zheng et al., 2019 [139]	Oxytocin [mean dose of IN-OT was 51 IU/day (range = 40–80 IU/day)]	As above	2–16 weeks duration (mean = 6.8 weeks; median = 6 weeks)	N = 9, n = 276	PANSS BPRS	No benefit NS [SMD = −0.04 (95% CI: −0.32, 0.24), *p* = 0.78; I^2^ = 17%]

## Abbreviations

AMPA: α-amino-3-hydroxy-5-methylisoxazole-4-propionic acid; BNSS: Brief Negative Symptom Scale; CDP-choline: cytidine 5′-diphosphocholine; CDSS: Calgary Depression Scale for Schizophrenia; COMT: Catechol-O-methyltransferase; COX: Cyclooxygenase; CRP: C-Reactive Protein; D2: Dopamine D2 receptors; DA: Dopamine; DB/RCT: Double-Blind Randomized Controlled Trial; DIEPSS: Drug-Induced Extrapyramidal Symptoms Scale; GAD: Glutamic acid decarboxylase; 5-HT: 5-hydroxytryptamine receptors; IL: Interleukin; IFN: Interferon; MAO: Monoaminoxidase; MoA: Mechanism of Action; NAC: N-acetylcysteine; nACh: nocotinic Acetylcholine-receptor; NaSSA: Noradrenergic and Specific Serotonergic Antidepressant; NDRI: Noradrenalin and Dopamine Reuptake Inhibitor; NE: Norepinephrine; NF-κβ: Nuclear Factor-κβ; NMDA: N-methyl-D-aspartate; NRI: Norepinephrine Reuptake Inhibitor; NS: Negative Symptoms; NSA-16: Negative Symptoms Assessment Scale-16 items; PANSS: Positive and Negative Syndrome Scale; PDE: Phosphodiesterase; PFC: Prefrontal Cortex; RCT: Randomized Controlled Trial; SANS: Scale for the Assessment of Negative Symptoms; SARI: Serotonin Antagonist and Reuptake Inhibitor; SER: Serotonin; SNRI: Serotonin-Norepinephrine Reuptake Inhibitor; SSRI: Selective Serotonin Reuptake Inhibitor; TCA: Tricyclic Antidepressants; TNF: Tumor Necrosis Factor.
